# Aligned CuO nanowire array for a high performance visible light photodetector

**DOI:** 10.1038/s41598-022-06031-y

**Published:** 2022-02-10

**Authors:** Min-Seung Jo, Hyeon-Joo Song, Beom-Jun Kim, Yoo-Kyum Shin, Sung-Ho Kim, Xu Tian, Sang-Min Kim, Min-Ho Seo, Jun-Bo Yoon

**Affiliations:** 1 School of Electrical Engineering, Korea Advanced Institute of Science and Technology (KAIST), 291 Daehak-ro, Yuseong-gu, Taejon, 34141 Republic of Korea; 2 SAMSUNG ELECTRONICS Co., Ltd., 1, Samsungjeonja-ro, Hwaseong-si, Gyeonggi-do 18448 Republic of Korea; 3 Department of Information Convergence Engineering, College of Information and Biomedical Engineering, Pusan National University, Yangsan, 50612 Republic of Korea; 4 Department of Micro and Nanosystems, KTH Royal Institute of Technology, Brinellvägen 8, 114 28 Stockholm, Sweden; 5 School of Biomedical Convergence Engineering, College of Information and Biomedical Engineering, Pusan National University, Yangsan, 50612 Republic of Korea

**Keywords:** Electrical and electronic engineering, Optical materials and structures, Nanoscale devices, Nanoscale materials, Nanowires

## Abstract

Recently, copper oxide (CuO) has drawn much attention as a promising material in visible light photodetection with its advantages in ease of nanofabrication. CuO allows a variety of nanostructures to be explored to enhance the optoelectrical performance such as photogenerated carriers scattering and bandgap engineering. However, previous researches neglect in-depth analysis of CuO’s light interaction effects, restrictively using random orientation such as randomly arranged nanowires, single nanowires, and dispersed nanoparticles. Here, we demonstrate an ultra-high performance CuO visible light photodetector utilizing perfectly-aligned nanowire array structures. CuO nanowires with 300 nm-width critical dimension suppressed carrier transport in the dark state and enhanced the conversion of photons to carriers; additionally, the aligned arrangement of the nanowires with designed geometry improved the light absorption by means of the constructive interference effect. The proposed nanostructures provide advantages in terms of dark current, photocurrent, and response time, showing unprecedentedly high (state-of-the-art) optoelectronic performance, including high values of sensitivity (*S* = 172.21%), photo-responsivity (*R* = 16.03 A/W, λ = 535 nm), photo-detectivity (D^*^ = 7.78 × 10^11^ Jones), rise/decay time (*τ*_*r*_/*τ*_*d*_ = 0.31 s/1.21 s).

## Introduction

Recently, visible light photodetectors have gained significant attention in scientific and industrial fields for a wide range of applications from traditional imaging, military monitoring, and optical communication to emerging technologies including environmental, biomedical, and wearable electronics^1–4^. As the field of application expands further, photodetectors with more functionality, such as transparency, flexibility, and broad absorption spectrum are required. Therefore, a wider range of materials including organic materials^5,6^, organic–inorganic hybrids^7^, quantum dots^8,9^, 2D nanomaterials^10,11^, and metal oxides^12–14^, have drawn attention in recent years as next-generation photodetector materials. Among various candidates, copper oxide (CuO), which has a high optical absorption coefficient and the lowest band gap energy among metal oxides, is a promising material for visible light photodetectors with transparency and flexibility^15–18^. In particular, the structure dependent optoelectrical characteristics of CuO, for example, its bandgap range and surface depletion layer for charge capturing, are controllable for suitable applications thanks to the material’s ease of nanofabrication. This feature enables a broad range of applications, such as multispectral detection and molecular/thermal imaging. Moreover, the intrinsic chemical/mechanical stability and cost-effectiveness of CuO are attractive strengths that will lead to reliable devices even for industrial applications. In this regard, numerous studies have been performed to exploit CuO for photodetectors^17–31^. Nonetheless, the electrically conductive characteristics of CuO, based on intrinsic hole accumulation at the surface, cause high dark current (*I*_*dark*_), resulting in unacceptably low sensitivity (*S*), photo-responsivity (*R*), and photo-detectivity (D^*^).

To improve the performance of CuO photodetectors, researchers have recently attempted to use CuO as a form of nanomaterial, such as nanoparticles (NPs) and nanowires (NWs). As the size of CuO is reduced to the nanometer scale, electrical resistivity can be dramatically increased due to the enhanced electron scattering, which favorably suppresses *I*_*dark*_ and enhances optoelectronic performance^19,21,22,32^. However, attempts to improve the performance of CuO with conventional nanomaterial approaches have been limited; previous works have simply utilized CuO nanomaterials made by synthesis and dispersion, resulting in randomly dispersed CuO nanomaterials^19,20,29^. Unfortunately, these random CuO nanomaterials make irregular and undesignable networks, creating an additional current pathway and increasing *I*_*dark*_. More importantly, these conventional nanomaterial methods limit the geometric design of nanomaterials, which is important for achieving additional performance enhancements in optical and photonic applications based on surface resonance^33–36^. Arbitrary shape also makes it difficult for CuO photodetectors to achieve uniform and reproducible device performance, which is crucial for practical usage of CuO. Thus, a transition of research viewpoint that can lead to geometrically and structurally designable nanomaterials are necessary for high-performance CuO photodetectors. Emerging nanofabrication technologies now enable us to fabricate multi-dimensional and scalable nanomaterials with high degrees of geometrical and structural design. The resulting geometrically and structurally designed nanomaterials have shown unprecedented performance in diverse device-level applications^37,38^. Moreover, spatially designed nanomaterials have guaranteed device-scalability, uniformity, reproducibility, and high-throughput, which are essential to their use in devices at an industrial level. Yet, there have been no studies that have demonstrated finely designable CuO nanomaterials for high-performance visible light photodetectors.

Here, we developed a high-performance visible light photodetector that utilizes a geometrically structured CuO nanowire (NW) array. To demonstrate the performance-enhanced CuO photodetector, we proposed a 3-D nanostructure that consists of a perfectly-aligned, dense CuO NW array on a silicon dioxide (SiO_2_) nanograting substrate. The proposed nanostructure can be ideal for maximizing and minimizing photocurrent (*I*_*ph*_) and *I*_*dark*_, respectively, because a perfectly-aligned NW array not only causes a dramatic reduction in electrical resistivity, but also enhances light absorption based on constructive interference by scattered light. We optimized the geometry of the proposed photodetector using finite-difference time-domain (FDTD) method. Then, to experimentally confirm the performance enhancements, we evaluated various photodetector devices with diverse shapes of CuO prepared using the developed nanofabrication methods. A parametric analysis of the fabricated device verified the dramatic enhancement in optoelectronic performance of the perfectly-aligned CuO NW component. The fabricated devices showed high values of *S*, *R*, and D^*^, and fast response time (*τ*); to the best of our knowledge, these values are at state-of-the-art level among CuO-based photodetectors. Finally, by using the formulated fabrication method, we successfully demonstrated tens of devices in an array on a cm-scale substrate, all of which showed high uniformity and stable optoelectronic characteristics.

## Results and discussion

**Figure 1:**
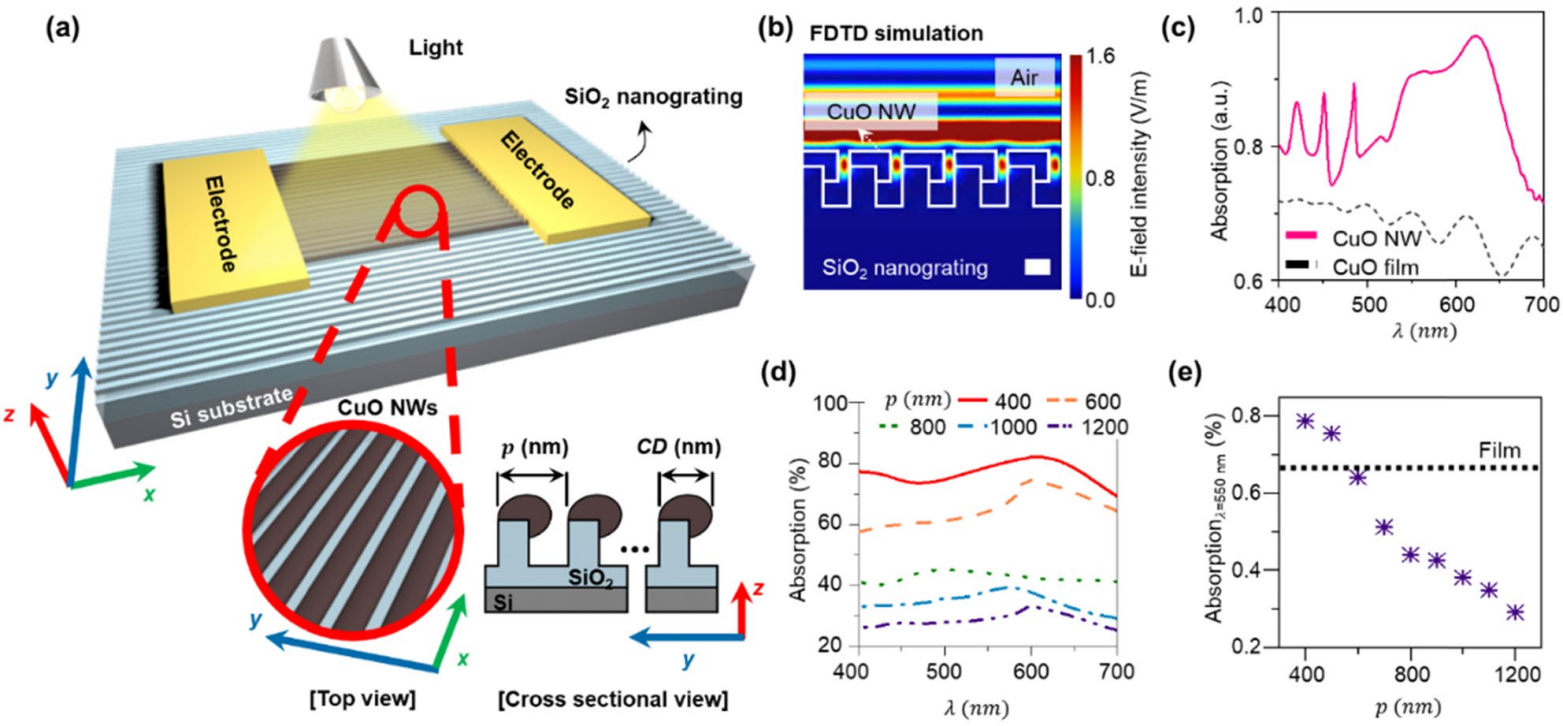
Concept of the proposed high-performance visible light photodetector. (**a**) 3-D schematic of the proposed device. The device is composed of the perfectly aligned CuO nanowire array (see [Top view]) on the SiO_2_ nanograting substrate (see [Cross sectional view]). (**b**) FDTD simulation result of the proposed device (scale bar = 200 nm). (**c**) Calculated absorption of the 400 nm-pitch (*p*) of CuO nanowire (NW) array on SiO_2_ nanograting substrate (pink solid line). Black dashed line indicates the absorption of CuO thin film (*thickness* = 122 nm). Calculated absorption varying pitch of CuO NWs with same dimension on SiO_2_ nanograting from 400 to 1200 nm (*width* = 314 nm, *thickness* = 114 nm): (**d**) visible light range (*λ* = 400–700 nm) (**e**) *λ* = 550 nm.

Figure 1a shows a schematic of the proposed photodetector. The proposed sensor is composed of a perfectly-aligned CuO NW array on an SiO_2_ nanograting substrate. There are two gold (Au) electrodes at both ends of the NWs where the optoelectronic characteristics are measured. Based on these geometrical structures, the proposed photodetector can exhibit high-optoelectronic characteristics because of the size-effect of the confined nanoscale and the enhanced light scattering in geometrically designed shape. High optoelectrical characteristics include (1) high sensitivity, (2) reduced response time, and (3) high responsivity, as follows. (1) Higher sensitivity (*S* = (*I*_*light*_ − *I*_*dark*_)/*I*_*dark*_)) by suppressed *I*_*dark*_: since the CuO NW has a nanometer scale cross-sectional dimension, electron (or hole) scattering is frequently generated, resulting in dramatically increased electrical resistivity and reduced *I*_*dark*_. Note that we defined the CuO NW width as a critical dimension (*CD*). (2) Reduced *τ* by CuO NW-structure: because the *CD* and thickness of CuO are at nanometer scale, the hole-electron recombination energy barrier (*Φ*) can be small. Small energy barrier results in active recombination of photo-generated carriers. Because longer response time is caused by subsequent slow diffusion of free carriers to the CuO surface, rapid recombination prevents current change by generated free carriers. (3) Higher responsivity (*R* = *I*_*ph*_/(*P*_*in*_ · *A*); *P*_*in*_ and *A* are power of incident light and light receiving area, respectively) and higher detectivity with higher *I*_*ph*_: because the CuO NWs are perfectly aligned with a certain pitch, the NWs do not form unnecessary networks with each other, resulting in an ideal light receiving field without any shaded areas. In particular, the periodic NW array can improve absorption based on the light scattering effect. As light enters the perfectly aligned and periodic CuO NW array, it is scattered at the surface of the NWs. At this moment, constructive or destructive interference by scattered light can form between the NWs depending on the geometry of the 3-D nanostructure; thus, optimized constructive interference can be induced by designing the shape and pitch of the CuO NW array, resulting in an increase of light absorption and higher *I*_*ph*_.

To optimize the geometry of the nanostructure to maximally enhance the absorption, we utilized an FDTD simulation to design the geometrical arrangement of the NW array. In the simulation, we first established the CuO NW array on the SiO_2_ nanograting structure; the thickness (*t*) of CuO NW was 114 nm, width (*w*), height (*h*), and pitch (*p*) of SiO_2_ nanograting are 200, 300, and 400 nm, respectively. It is worthwhile to note that we also performed FDTD simulation of a CuO thin film of *t* = 122 nm with the same unit volume as the NWs to fairly compare the nanostructure effect of the proposed device. Details about the FDTD simulation are included in Supplementary Fig. S1. The calculated E-field intensity in visible light wavelength range (*λ* = 400–700 nm) is shown in Fig. 1b. Comparing the E-field intensity of the CuO film (Supplementary Fig. S2), notable E-field concentration is additionally generated in the space between the CuO nanowires (red in Fig. 1b). We can expect that the E-field concentration between the nanostructure causes more light to be absorbed through the sides of the CuO NW in the overall visible light range (*λ* = 400–700 nm). Indeed, we confirmed from the simulation results that the light absorption of a nanowire is significantly higher than that of a thin film in the overall visible light range (Fig. 1c), implying the better optoelectronic characteristics of the CuO NW device. With the theoretical confirmation of the proposed concept, it is then essential to optimize the structure of the CuO NW array for maximized performance.

In a periodic structure of CuO NWs, the pitch (*p*) value is important to optimize the optical performance. This affects not only the light interference, but also the fill factor of the CuO NWs, which is important in determining the effective light receiving area of CuO in the entire exposed area. This effective area ultimately determines the amount of absorption of the proposed 3-D nanostructure. The simulation was performed with various values of *p* of the CuO NW array on SiO_2_ nanograting, from 400 to 1200 nm, having the same geometry of width and thickness (*w* = 314 nm and *t* = 114 nm) of CuO NW and height (*h* = 300 nm) of SiO_2_ nanograting structure (Fig. 1d). In the calculated visible light range (*λ* = 400–700 nm), the amount of absorption from the CuO nanowire array on the SiO_2_ nanograting substrate increases as *p* decreases. The absorption of light at 550 nm is plotted with respect to *p* for quantitative comparison (Fig. 1e). The absorption continuously increases as *p* becomes smaller from 1200 nm; then, a dramatic increase of absorption is generated as *p* becomes smaller than 800 nm. Although there is a loss of absorption area due to the gap in which NW is separated, the amount of absorption from the nanowires still exceeds that of a film when *p* is smaller than 500 nm. This phenomenon is mainly due to constructive interference resulting from the high fill-factor of the CuO nanowires. The maximum absorption at *p* = 400 nm is ~ 0.8, which is 115% of that of the film, implying a significant improvement in optoelectronic performance through higher degree of light absorption due to the smaller dimensions of nanoscale. As shown in Supplementary Fig. S3, the intensity of the E-field can fluctuate with respect to varying *p* because of constructive and destructive interference between the structures.

**Figure 2:**
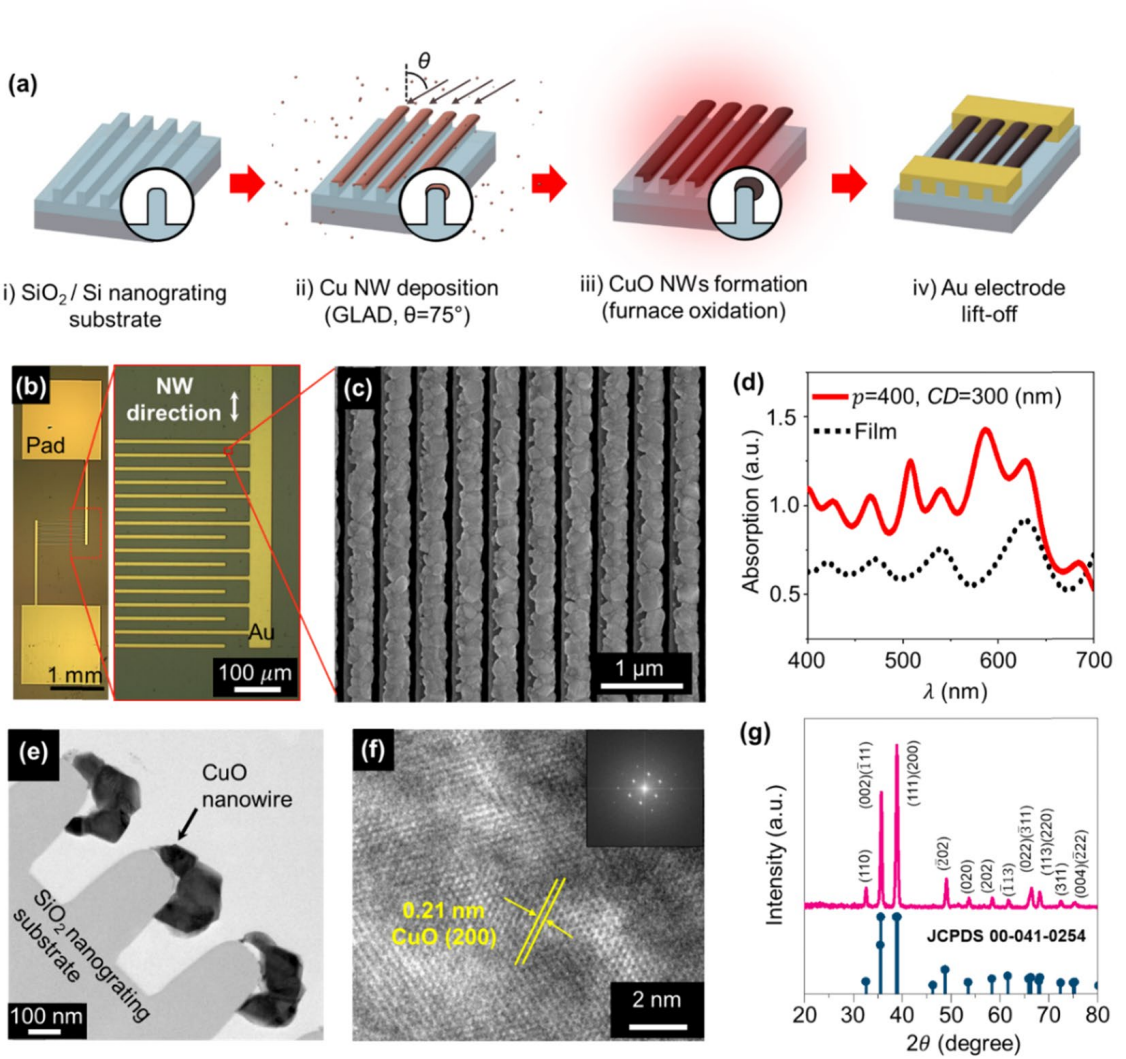
Device demonstration. (**a**) 3D-schematics of the fabrication process. (**b**) Optical image of the fabricated device (left panel). The CuO nanowires are fabricated on the entire area on the substrate. The red dashed box is magnified in the right panel. (**c**) Top SEM image of the fabricated CuO nanowires. (**d**) The measured absorption data of the fabricated CuO nanowires (red solid line) and thin film CuO (black dashed line). (**e**) TEM HR mode of fabricated CuO nanowire (cross-sectional view). (**f**) Magnified TEM image of CuO NW (inset: Fourier transform pattern of single grain of CuO). (**g**) The measured XRD pattern of the fabricated CuO nanowire (upper panel) and reference diffraction lines of the monoclinic tenorite phase of CuO (JCPDS 00-241-0254) (lower panel).

Next, we developed a fabrication process that enables us to experimentally confirm the enhanced absorption of the proposed 3-D CuO NW array. The fabrication started with a pre-designed SiO_2_ nanograting substrate (Fig. 2a,i); the SiO_2_ nanograting was manufactured from Si nanograting via wet-chemical size reduction process^39^ and thermal oxidation. The process of preparing the substrate and a scanning electron microscope image of the substrate are shown in Supplementary Fig. S4. The perfectly-aligned NWs were then easily fabricated on the SiO_2_ nanograting substrate by glanced angled deposition (GLAD) method (*θ* = 75°) (Fig. 2a,ii). Since the Cu was deposited on the 3-D nanograting substrate at a specific angle, the 1-D NW of Cu can be formed on the top of the nanograting by shadowing effect. After deposition, the Cu NW was oxidized at a high temperature (700 °C for 3 h in air ambient) to become CuO NW (Fig. 2a,iii). Finally, Au electrodes (*thickness* = 150 nm) are formed on the CuO nanowires using a conventional lift-off process (Fig. 2a,iv). The details of device fabrication are included in the *Methods* section. Because all processes including the fabrication of the Si nanograting substrate are compatible with conventional semiconductor manufacturing processes, the proposed nanowire fabrication method is economically advantageous for reliable device fabrication. Visual inspection on the fabricated device was performed using an optical microscope (Fig. 2b) and SEM (Fig. 2c). The CuO NW array was formed on the entire area of the SiO_2_ nanograting substrate, and the interdigitated shape of the Au electrodes establishes an electrical current pathway through the nanowires in the length-direction. The uniformly isolated CuO nanowire array without any interconnection between the NWs can be confirmed in the SEM image, which indicates perfectly-aligned CuO NWs in the width-direction (Fig. 2c). With the fabricated device, we also confirmed, via visible light spectrometer measurements, an enhanced light absorption characteristic of the fabricated CuO NW arrays compared to the CuO film (Fig. 2d). The absorption of the CuO NW array is higher than that of a CuO film in overall visible-light range; the results are also highly coincident with the simulation results shown in Fig. 1c.

To understand the material quality of the fabricated CuO, material analysis was conducted using TEM, X-ray diffraction (XRD) analysis, energy dispersive X-ray spectroscopy (EDS), and X-ray photoelectron spectroscopy (XPS). The cross-sectional TEM image of the CuO NWs confirmed that the CuO nanowires have a granular structure (Fig. 2e). From the TEM image, we also confirmed that the nanowires are formed only on the top of the SiO_2_ nanograting, as we designed. Using high-resolution (HR) mode and Fourier transform pattern of the TEM, it was confirmed that each grain had a high quality single crystalline structure (Fig. 2f). The crystallinity of the overall CuO NWs was further evaluated using XRD analysis. The measured XRD data showed that the crystalline CuO NWs highly matched the diffraction lines of the monoclinic tenorite phase of CuO (JCPDS 00-041-0254) (Fig. 2g). EDS material mapping analysis from the TEM image showed high purity of the CuO nanowires, without any significant material diffusion or degradation (Supplementary Fig. S5). In the XPS measurement results of the chemical-state, CuO also highly corresponded with the conventional peaks of pure CuO (Supplementary Fig. S6). By investigating the material properties of CuO NW, we confirmed that our fabrication method can successfully produce perfectly-aligned CuO nanowires with high material quality and chemical stability.

**Figure 3:**
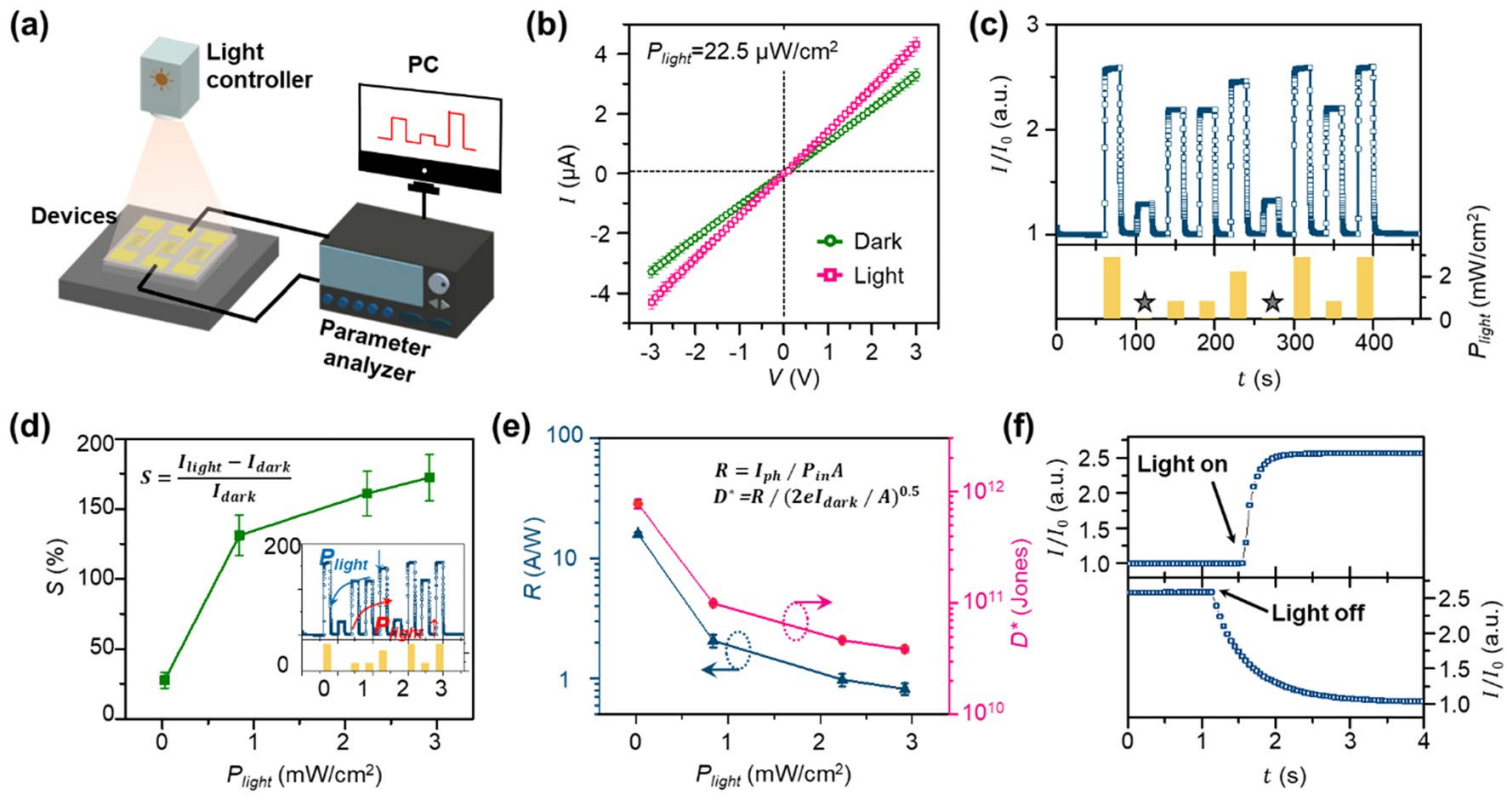
Characterization of the fabricated photodetector. (**a**) Schematics of the measurement set-up. (**b**) The measured *I–V* characteristics of the device (green circle: dark state, pink square: *P*_*light*_ = 22.5 μW/cm^2^). (**c**) The dynamic optoelectronic response of the fabricated photo-detector (upper panel), and the intensity of incident light (gray solid star: *P*_*light*_ = 22.5 μW/cm^2^). (**d**) The calculated sensitivity (*S*) of the device with respect to various *P*_*light*_ (inset: measured *S* changes by increasing and decreasing *P*_*light*_). **e)** The calculated photo-responsivity (*R*) and photo-detectivity (D^*^) of the device with respect to various *P*_*light*_ . **f)** The dynamic response of the photodetector for *P*_*light*_ = 22.5 μW/cm^2^; rise time (*τ*_*r*_) and decay time (*τ*_*d*_) were 0.329 s and 1.21 s, respectively.

Next, we performed optoelectronic characterization of the fabricated device. For the measurement, we established a customized visible light optical set-up using a commercial halogen lamp with parameter analyzer (Fig. 3a). Details of the measurement set-up and information on the light source are included in the *Methods* section and Supplementary Fig. S7, respectively. First, the current–voltage (*I–V*) characteristics of the device in dark state (the green circle in Fig. 3b) and lighted condition (*P*_*light*_ = 22.5 μW/cm^2^, the pink square in Fig. 3b) were measured using three devices with identical dimensions. From the ohmic contact behavior, we performed subsequent optoelectrical measurements at under 3 V (*I*_*dark*_ = 3.29 μA). To examine the dynamic optoelectronic response of the device at various *P*_*light*_, the normalized *I* and incident *P*_*light*_ changes with respect to time are plotted in the upper and lower panels of Fig. 3c, respectively. As *P*_*light*_ dynamically changed to 22.5 μW/cm^2^, 0.840 mW/cm^2^, 2.24 mW/cm^2^, and 2.92 mW/cm^2^, normalized *I* changed to 4.24, 7.21, 8.10, and 8.52 μA, respectively. Using an optoelectronic response test and randomly changing *P*_*light*_ (Fig. 3c), we also confirmed that the device showed reproducible and reliable optoelectronic characteristics. To quantitatively understand the optoelectronic characteristics of the proposed device, we evaluated its sensitivity (*S* = *I*_*ph*_/*I*_*dark*_), photo-responsivity (*R* = *I*_*ph*_/(*P*_*in*_ · *A*)), and detectivity (D^*^ = *R*/(2*eI*_*dark*_/*A*)^0.5^), where *I*_*ph*_ is photocurrent (*I*_*ph*_ = *I*_*light*_ − *I*_*dark*_), *A* is the illuminated area, and *e* is 1.6 × 10^–19^ *C*. It should be noted that *A,* representing the exposed area, including nanowire array and the substrate, between the interdigitated electrodes, was 0.0024 cm^2^. Figure 3d shows the measured *S*. When the light intensity was 22.5 μW/cm^2^, the measured sensitivity was 27.50%. *S* increased to 131.08, 160.85, and 172.21%, when *P*_*light*_ increased to 0.840 mW/cm^2^, 2.24 mW/cm^2^, and 2.92 mW/cm^2^, respectively. In the calculation, *P*_*light*_ is measured around the 535 nm wavelength spectrum. The increase in *S* with higher intensity can be explained as the result of more free carriers being induced by the higher *P*_*light*_. Notably, the generated photocurrent was not linear. As the light intensity increases, photo-generated electron–hole pairs are saturated; as a result, excess photons cannot be absorbed. More importantly, the proposed device showed negligible hysteresis during increasing and decreasing of the light intensity (inset in Fig. 3d). We believe that this result originates from the suppressed slow oxygen desorption reaction. In bulk CuO, oxygen can diffuse into the bulk and then generate an additional desorption reaction with the electrons induced by the light. However, the nanowire intrinsically has a small volume and most of the CuO parts initially fully react with oxygen because of the large surface-to-volume ratio. Thus, little additional oxygen desorption is generated in the nanowire. Next, we calculated values of *R* and D^*^ of the device (Fig. 3e). The calculated *R* and D^*^ were 16.03 A/W and 7.78 × 10^11^ Jones, respectively, at *P*_*light*_ of 22.5 μW/cm^2^. The *R* and D^*^ decreased as *P*_*light*_ increased; at *P*_*light*_ = 0.840 mW/cm^2^, 2.24 mW/cm^2^, and 2.92 mW/cm^2^, *R* = 2.07, 0.98, and 0.82 A/W and D^*^ = 9.98 × 10^10^, 4.66 × 10^10^, and 3.86 × 10^10^, respectively. The decreases in *R* and D^*^ can also be explained by the saturated depletion of the photo-generated electron–hole pairs under high *P*_*light*_. To evaluate the response time, we quantitatively measured *τ*, which was defined as the time consumed to generate 90% change in *I* by applied *P*_*light*_. *I* changes induced by *P*_*light*_ = 2.92 mW/cm^2^ were measured and plotted with an extended time axis (Fig. 3f). When the light was turned on and off, the photodetector showed an immediate *I* change, and the measured rise time (*τ*_*r*_) and decay time (*τ*_*d*_) were 0.329 s and 1.21 s, respectively.

**Figure 4:**
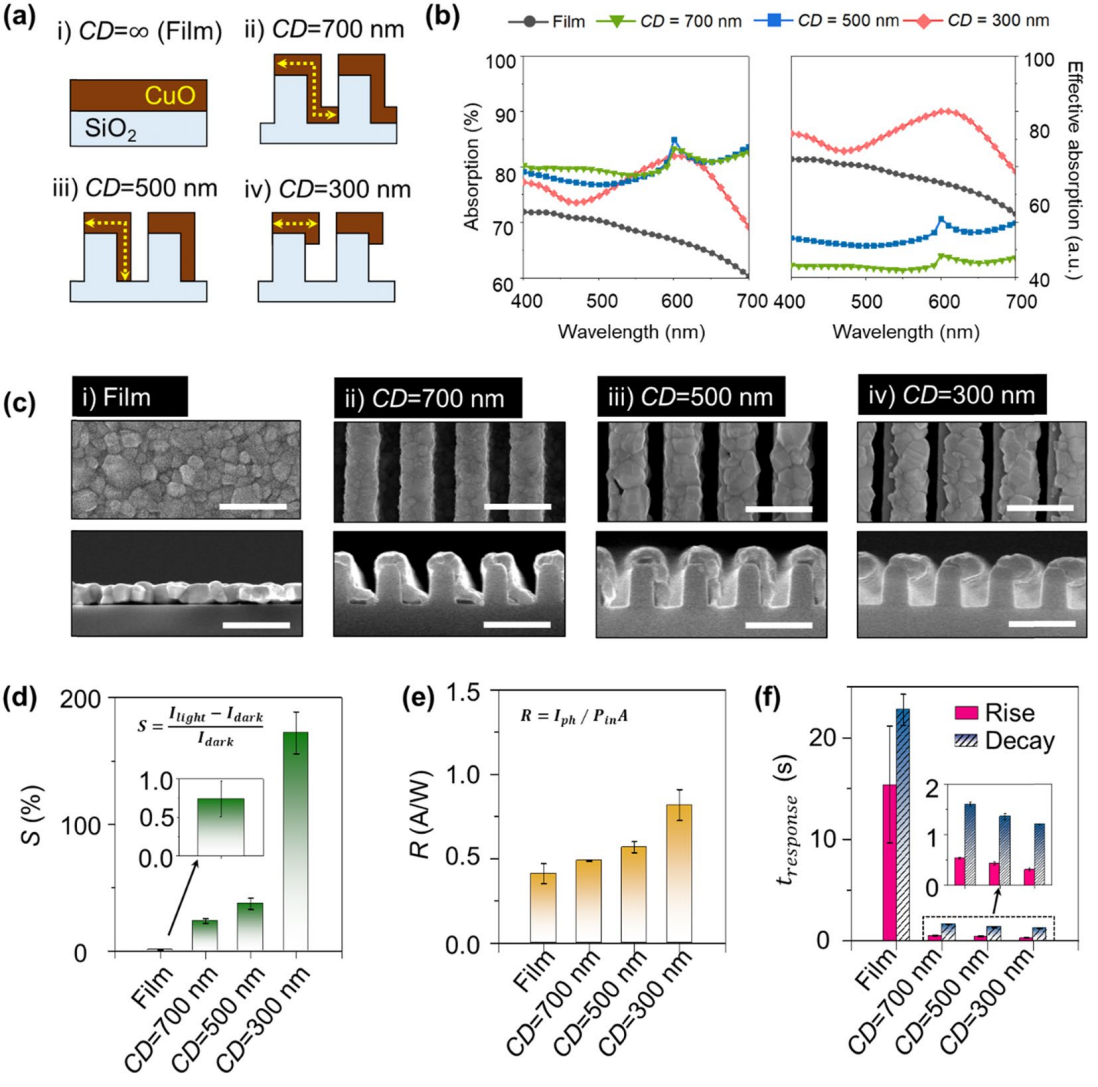
Performance comparison of various device shapes with different critical dimensions (*CD*). (**a**) Top (upper panel) and cross-sectional (lower panel) SEM image of various fabricated CuO shapes. (**b**) Calculated absorption of NW array with different *CDs*. (left panel: calculated value, right panel: normalized value using unit volume of CuO). (**c**) Fabricated CuO film and CuO NWs with different *CDs* (upper panel: top view, lower panel: cross-sectional view/scale bar 500 nm). Calculated optoelectrical results in comparison to different CuO nanostructures having different *CD,* (**d**) sensitivity (*S*), e) photo-responsivity (*R*), and (**f**) response time of the fabricated samples. All data are calculated from the optoelectronic response for *P*_*light*_ = 22.5 μW/cm^2^.

To clarify the high optoelectronic performance of the proposed photodetector, we further investigated the optoelectronic performances of various photodetectors with perfectly-aligned CuO NWs on SiO_2_ nanograting with different shapes. Specifically, the *CD* of the CuO NWs are varied: (1) film (*CD* = ∞), (2) *CD* = 700 nm, (3) *CD* = 500 nm, and (4) *CD* = 300 nm (Fig. 4a). It is noteworthy that CD is an appropriate design parameter to optimize the higher optoelectronic performances. Not only *CD* is critically associated with *I*_*dark*_, but it can also determine the light-incident area and scattering effect, resulting in different light absorption. Before the experimental study, we calculated the absorption of each case using FDTD. In the visible light spectrum (*λ* = 400–700 nm), each case shows different absorption (Fig. 4b). While the film type CuO shows noticeably lower absorption, the CuO nanostructures present higher absorption. This result can be explained as the result of the enhanced light absorption by scattering at the surface of the CuO nanostructures, as shown in Fig. 1b. To evaluate the effective absorption of the various CuO nanostructures, we further performed normalization of light absorption of each case using unit volume of CuO. Since the optoelectronic performance of the device is determined by the variation of the optoelectronic response of the entire CuO volume, it is reasonable to consider only the amount of light absorbed at unit CuO nanostructures. Thus, we calculated the effective absorption of each CuO nanostructure by dividing the absorption with the volume of CuO. From the calculated results, we can confirm that the highest effective absorption can be achieved for the *CD* = 300 nm CuO structure, and we can explain this phenomenon by referring to the maximized E-field concentration induced by the structure (Supplementary Fig. S8).

To experimentally confirm the design study, we further fabricated photodetector devices having different *CD* of CuO (Fig. 4c). The various *CDs* of the NW are fabricated by controlling the deposition angle. The 700, 500, and 300 nm CuO NW *CDs* are fabricated by depositing identical thicknesses of 40 nm of Cu by GLAD method with 75°, 45°, and 23° angles, respectively, and then performing oxidation (700 °C in air). First, we measured the *I–V* characteristics of the devices in a dark state. From the measured *I–V* curves of the devices, a noticeable variation in electrical resistance was observed with respect to the *CD* values of the NWs. To clearly compare the electrical resistances of the different nanowires, we extracted the sheet resistance of each device in a dark state (Supplementary Fig. S9). At 3 V, the sheet resistance of the film sample was 3.9 Mohms/sq. However, the sheet resistances of other photodetector samples significantly increased as the *CD* of CuO decreased; the measured resistances were 120.4, 135.0, and 211.8 Mohms/sq when the *CD* values were 700, 500, and 300 nm, respectively. The increase in resistance can be explained by the decrease of *CD* by the enhanced electron scattering in the nanowire structure. As many studies have previously reported, electron scattering significantly increases as the size of a conductive material decreases, resulting in increased resistance^19,21,22,32,40^. We further tested the dynamic optoelectronic characteristics of all samples. All devices were exposed to randomly different light, and the optoelectronic responses were measured (Supplementary Fig. S10). For the quantitative comparison, we first extracted *S* from the optoelectronic response of the samples to *P*_*light*_ = 2.92 mW/cm^2^. As the CuO NWs became smaller and *CD* decreased, an exceptionally higher *S* was achieved (Fig. 4d). Specifically, *S* was enhanced by more than 230 times with the *CD* = 300 nm nanowire compared to that of the film sample. With the CuO film sample (*CD* = ∞), the *S* was 0.74%, and *S* of the CuO NW samples increased to 23.69, 37.23, and 172.21%, as the *CD* decreased to 700, 500, and 300 nm. We attribute this dramatic enhancement in *S* with the *CD* = 300 nm device to the successful reduction in *I*_*dark*_ due to electron scattering, which occurred without degrading *I*_*ph*_. Next, we extracted the *R* of the samples (Fig. 4e). The *R* of the nanowire devices was also remarkably higher than that of the film device. The *R* of the film and of devices with *CD* = 700, 500, and 300 nm were 0.41, 0.49, 0.57, and 0.82 A/W, respectively. This represents a 2.0-times increase in the *CD* = 300 nm device compared to the film device. We believe this dramatic enhancement in nanostructure was obtained by scattering effect in the periodic nanostructure, as we have discussed in Fig. 1. Lastly, we compared the *τ* of the samples (Fig. 4f). *τ* was also obtained with *P*_*light*_ = 2.92 mW/cm^2^. For the CuO film sample, *τ*_*r*_ and *τ*_*d*_ were 15.40 and 22.76 s, respectively. In contrast, the nanowire samples achieved greatly reduced values of *τ*_*r*_ and *τ*_*d*_. Specifically, *τ*_*r*_ and *τ*_*d*_ were 0.54 and 1.61, 0.43 and 1.36, and 0.31 and 1.21 s for values of *CD* = 700, 500, and 300 nm, respectively. These improvements in *τ* according to the reduction in *CD* can be explained by the reduced conductive channel effect^41^. In the smaller *CD* nanowires, free carriers travel through a smaller area of effective conductive channel; the reduced scale of the effective conductive channel shortens the carrier transit time, which leads to faster *τ*.

**Figure 5:**
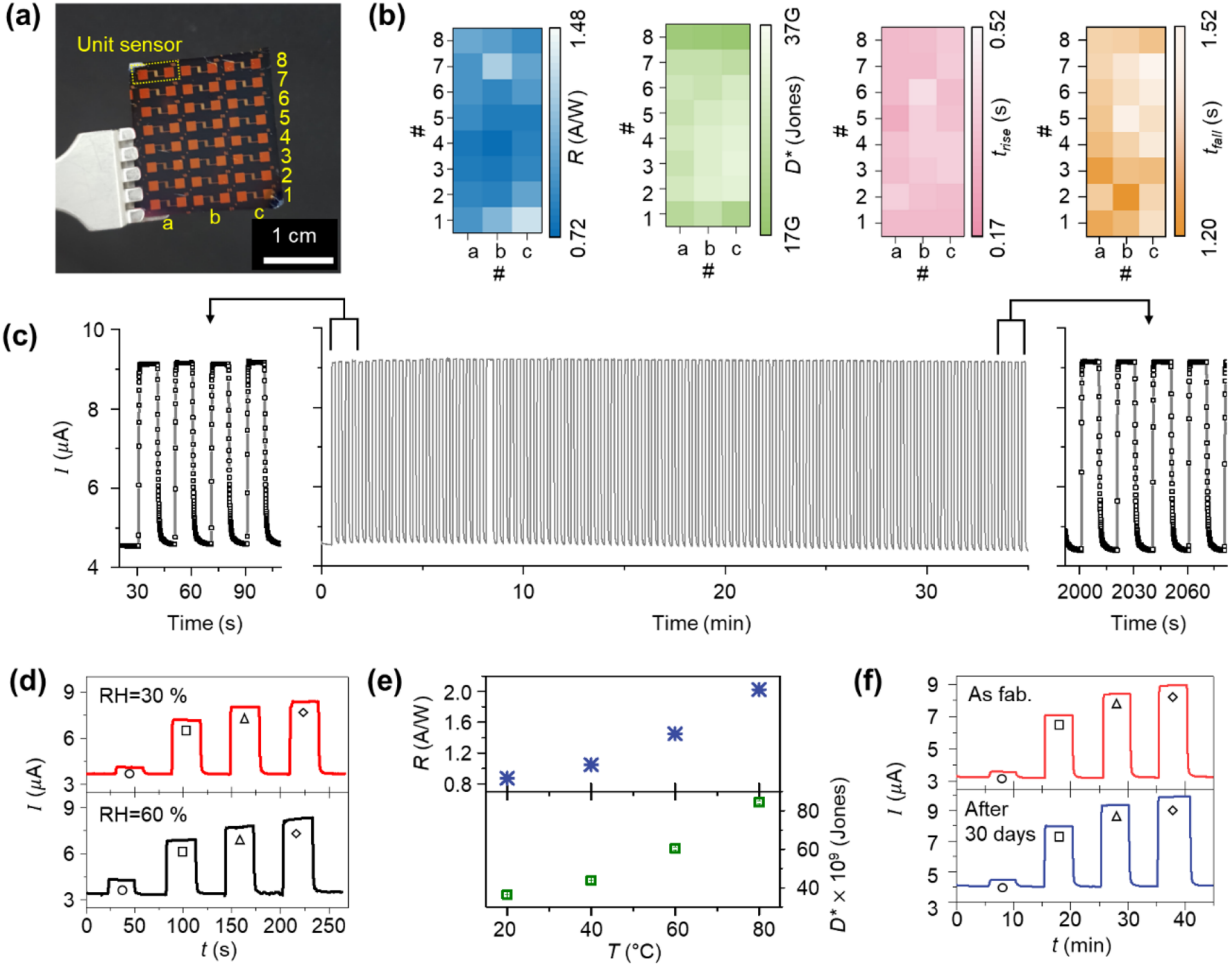
(**a**) Optical image of the fabricated 24-unit device array. (**b**) Photo-responsivity (*R*), photo-detectivity (D^*^), and rise (*τ*_*r*_) and decay (*τ*_*d*_) time of the fabricated device array. (**c**) 100-times repetitive illumination test with *P*_*light*_ = 2.92 mW/cm^2^. Performance stability of the device within various environmental conditions. (**d**–**f**) Stability of the fabricated device. Optoelectronic characteristics in different humidity conditions (**d**), and *R*, D^*^ changes of the device with respect to external temperature (**e**). Optoelectronic characteristic changes after 30 days (**f**).

So far, we have verified that the proposed perfectly-aligned CuO nanowire, based on its unique structural form-factor, enables a high-performance photodetector. To clearly understand the superior optoelectronic performances of the proposed device, we compared the performance of the device (*CD* = 300 nm) with those of other photodetectors. To the best of our knowledge, compared to recently developed CuO-based photodetectors (Table 1)^17–28,30,42^, the proposed device exhibited state-of-the-art levels of photo-responsivity, photo-detectivity, sensitivity, and response time. One further advantage of the proposed device is its compatibility with conventional semiconductor fabrication. All of the fabrication processes used for the device are conventional semiconductor processes, which helps to ensure device uniformity, reproducibility, and high-throughput. To highlight the advantages in fabrication, we fabricated a 24-device array and evaluated its performance uniformity (Fig. 5a). An optical image of the fabricated device array is shown in Fig. 5a. There was no significant device failure, and all devices showed relatively uniform optoelectronic characteristics (Fig. 5b). The measured *R*, D^*^, *τ*_*r*_, and *τ*_*d*_ values at light intensity of 2.92 mW/cm^2^ are shown in Fig. 5b; the average *R*, D^*^, *τ*_*r*_, and *τ*_*r*_ of all devices were 0.93 ± 0.6 A/W, 2.73 ± 0.45 × 10^10^ Jones, 0.35 ± 0.04 s, and 1.39 ± 0.08 s, respectively. In addition, we tested the operation stability of the fabricated device in repetitive illumination and various environmental conditions. The repetitive operation test was performed more than 100 times with an interval of 30 s at a light intensity of 2.92 mW/cm^2^. The result shows that *I*_*light*_ and *I*_*dark*_ are consistent with the initial values even after the 100th operation (Fig. 5c). Next, the relative humidity (RH) was changed from 30 to 60%; however, there was no significant change in optoelectronic performance (Fig. 5d). The device also showed almost identical optoelectronic responses with various incident lights (circle: 22.5 μW/cm^2^, square: 0.840 mW/cm^2^, triangle: 2.24 mW/cm^2^, and diamond: 2.92 mW/cm^2^). We believe this result can be attributed to the regular nano-structuring of the device; the device consists of a perfectly-aligned nanowire array, which prohibits surficial coating and coverage by water droplets. We also tested the effect of temperature on the device. As the external temperature increased, values of *R* and D^*^ of the device increased at the light intensity = 2.92 mW/cm^2^ (Fig. 5e). *R* and D^*^ of the device at 20, 40, 60, and 80 °C were 0.67 A/W and 31.81 × 10^9^ Jones, 0.80 A/W and 38.24 × 10^9^ Jones, 1.11 A/W and 52.84 × 10^9^ Jones, and 1.55 A/W and 73.85 × 10^9^ Jones, respectively. These increases in *R* and D^*^ can be explained by the enhancement in carrier generation induced by external temperature^43,44^. When thermal energy is applied to a semiconductor, additional free carriers, such as electrons and holes, can be generated, and more *I*_*ph*_ is induced. Even though temperature can induce a significant variation in performance, it can be compensated for in the future by incorporating an additional temperature sensor. Finally, we measured the change in device performance over time. The device was stored at room temperature condition for 30 days, but there was no significant change in optoelectronic response with incident light (Fig. 5f, circle: 22.5 μW/cm^2^, square: 0.840 mW/cm^2^, triangle: 2.24 mW/cm^2^, and diamond: 2.92 mW/cm^2^).

**Table 1:** Comparison of the performance of recently developed CuO photodetectors.

References	Structure	Light source/applied voltage	Responsivity [A/W]	Detectivity [Jones]	Response time (rise/fall)	Sensitivity (%)	Fabrication process
**This work**	**Perfectly- aligned nanowire array**	**Visible light (400–700 nm)/3 V**	**16.0 A/W (*P***_***in***_** = 22.5 **${\varvec{\mu}}$** W/cm**^**2**^**)**	**7.78 **$\times$** 10**^**11**^** (*P***_***in***_** = 22.5 **${\varvec{\mu}}$** W/cm**^**2**^**)**	**0.31 s/1.21 s**	**172.2 (*P***_***in***_** = 2.92 mW/cm**^**2**^**)**	**Semiconductor batch-process**
*Appl. Surf. Sci.* 2015, *346,* 18–23	Film	Visible light (520 nm, *P*_*in*_ = 1 mW/cm^2^)/2 V	~ 11 $\times$ 10^–3^	N/A	N/A/ < 1 $\mu s$	~ 3	Physical vapor deposition
*Superlattice. Microstruct.* 2018, *113,* 754–760	Film	Visible light (650 nm, *P*_*in*_ = 5 mW/cm^2^)/5 V	5.9 $\times$ 10^–4^	4.60 $\times$ 10^8^	52.28 s/44.2 s	57.5	Physical vapor deposition
*Sci. Rep.* 2019, 9, 7334	Film	Visible light (400–700 nm)/3 V	15.3 A/W (*P*_*in*_ = 22.5 $\mu$ W/cm^2^)	1.08 $\times$ 10^11^ (*P*_*in*_ = 22.5 $\mu$ W/cm^2^)	0.682 s/1.77 s	4.06(*P*_*in*_ = 0.840 mW/cm^2^)	Semiconductor batch-process
*IEEE Electron Device Lett*. 2017, 39. 1. 47	Film	Visible light (633 nm, *P*_*in*_ = 500 mW/cm^2^)/− 20 V	0.17	1.38 $\times$ 10^11^	0.48 s/0.53 s	33.3	Sol–gel
*IEEE Electron Device Lett.* 2018, 40. 2. 255	Film	UV (245 nm, *P*_*in*_ = 2.11 mW/cm^2^)/− 20 V	7.77	3.08 $\times$ 10^11^	N/A	820	Solution process and thermal oxidation
*Appl. Surf. Sci.* 2018, *452,* 155–164	Dispersed nanoparticle	UV-NIR (*P*_*in*_ = *AM*1.5G)/2 V	5.4	3.28 $\times$ 10^10^	16 ns	45	Synthesis
*J. Phys. Chem. C* 2010, *114,* 2440–2447	Single nanowire	Visible light (540 nm, *P*_*in*_ = 1 mW/cm^2^)	~ 8	N/A	N/A/ ~ 36 s	87 (*P*_*in*_ = 45 mW/cm^2^)	Synthesis and transfer
*Mater. Lett.* 2014, *117,* 217–220	Dispersed nanowires	Visible light (405 nm, *P*_*in*_ = 11.1 mW/cm^2^)	N/A	N/A	N/A/0.45 s	4	Thermal oxidation
*Nanoscale Res. Lett.* 2014, *9,* 637	Single nanowire	Visible light (600 nm*, P*_*in*_ = 2 mW/cm^2^)/5 V	200	6.38 $\times$ 10^11^	N/A	1.35	Electrochemical
Appl. Phys. Lett. 2020, 116, 111102	Nano-semiparaboloids	UV-NIR (250–900 nm, *P*_*in*_ = 5 mW/cm^2^)/20 V	2.6 $\times$ 10^–3^	2 $\times$ 10^9^	N/A	1.25	Hydrothermal method
Small Methods 2021, 2100202	Microhemisphere-Nanowire	NIR (810 nm, *P*_*in*_ = 7.4 mW/cm^2^)/1 V	2.5 $\times$ 10^–7^	4.3 $\times$ 10^8^	18.3/144.9	1.3	Thermal oxidation growth
*ACS applied materials and interfaces* 2010, *2. 5.* 1536	Mesoporous dandelion	Visible light	N/A	N/A	46 s/29.8 s	N/A	Synthesis (hydrothermal method)
*Sens. Actuators, A* 2016, *239.* 209	Nano rod array	NIR (760–910 nm, *P*_*in*_ = 0.1 mW/cm^2^)/2 V	1.6	N/A	0.41 s/0.25 s	700	Cu synthesis (aqueous solution) and oxidation
*IEEE Electron Device Letters,* 2021*, 42.6: 875–878*	Nano-composite	UV-NIR (600 nm, *P*_*in*_ = 0.168 μW)/− 1 V	8.74	2.78 × 10^13^	12.16 μs/26.71 μs	3.75–55.16	Solution process and spin coating

## Conclusions

In conclusion, we have proposed a visible light photodetector with a perfectly-aligned CuO nanowire array on a 3-D structure. Electrons guided in the inherently narrow nanowires suppress *I*_*dark*_ and enhance *I*_*ph*_. In particular, the periodic characteristic of the perfectly-aligned CuO nanowires on nanograting substrate with high fill-factor contribute to absorption enhancement via constructive interference effect. Through the simulation of light absorption, and based on device’s geometric structure, we optimized the dimensions of the perfectly-aligned CuO NWs on a nanograting substrate with values of *p* = 400 nm and *CD* = 300 nm. To demonstrate the proposed device, we developed a nanofabrication method to easily fabricate a perfectly-aligned CuO nanowire array based on a conventional semiconductor process. The fabricated photodetector exhibited high optoelectronic performance, including values of *S* = 172.21%, *R* = 16.03 A/W, D* = 7.78 × 10^11^ Jones, and *τ*_*r*_/*τ*_*d*_ = 0.31/1.21 s. These values are the highest levels reported for CuO based photodetectors. We also confirmed that the proposed device can be operated with high stability at various levels of humidity (30–60% RH) and temperature (20–80 °C). The device exhibited excellent durability over 100 cycles of operation and its performance did not degrade even after 30 days in ambient environment. Finally, we demonstrated a 24-array photodetector with no significant device failure during fabrication; all devices showed great uniformity in values of *R*, D*, and *τ*_*r*_/*τ*_*d*_. The proposed method offers a guide in that the geometrical arrangement of nanomaterials is a forefront method for designing high-performance photodetector structures that consider light-matter interaction. The proposed concept also provides a valuable example of geometrically structured nanomaterials, which have recently attracted interest in the fields of materials and nanoengineering.

## Methods

### Fabrication of SiO_2_ nanograting substrate

The silicon (Si) nanograting substrate (150 nm-width and 200 nm-space) was first fabricated using KrF lithography. Then, the 200 nm-width nanograting was reduced to 90 nm-width by repetitive size reduction based on the Si surface oxidation and etching technique. This size reduction was conducted before Si oxidation, because significant volume expansion is generated by the oxidation process. Then, a wet oxidation process (140 min, 1100 °C in H_2_, O_2_ ambient) was conducted on the 70 nm-width Si nanograting substrate to form silicon dioxide (SiO_2_) on the surface. SEM images of the SiO_2_ nanograting substrate fabrication are shown in Supplementary Fig. S4.

### Fabrication of photodetector

Perfectly-aligned nanowires were fabricated by oblique PVD at different angles for different *CD*. To produce *CD* = 300, 500, and 700 nm devices, Cu was deposited with identical thicknesses (40 nm) at angles of 75°, 45°, and 23°, respectively. After deposition, CuO was formed by thermal annealing process (700 °C for 1 h in air atmosphere with heating rate of 10 °C/min and natural cooling). Photoresist (NR9-3000PY, Futurrex, Inc.) was applied to the substrate using a spin coating process (*thickness* = 2.2 μm, 4000 rpm, 40 s), where the CuO nanowire formed, to pattern the area of the electrode. A mask aligner (MJB4, SUS MicroTec) was used for the photolithography process. For the interdigitated electrodes with adhesion layer, 10 nm-thickness of chromium (Cr) and 150 nm-thickness of gold (Au) were deposited on the CuO patterned substrate using PVD. Finally, the photoresist was removed in acetone solution for 1 min. during sonication. Fabrication details are shown in Supplementary Fig. S11.

### Characterization of the photodetector

The measurement set-up was established with a probe station chamber, commercial vacuum probe station (MST 5500B, MS tech), parameter analyzer (Keithley 2636B, Tektronix Co.), programmed personal computer, and lamp (halogen-Fok-100 W, Fiber Optic Korea Co., LTD) with intensity controller. For the measurement, the device was held on the probe station by vacuum, and Au electrical probes were gently connected to the Au electrodes of the device. For the dark state, the chamber was closed, and the area around the device was maintained in the dark. The spectrum of the light source is shown in Supplementary Fig. S6.

## Supplementary Information

Supplementary Information.
