# Supplementary material for: Aligned CuO nanowire array for a high performance visible light photodetector
Source: Sci Rep. 2022 Feb 10;12:2284. doi: 10.1038/s41598-022-06031-y (PMC8831480; doi:10.1038/s41598-022-06031-y)
Supplement: Supplementary file 1 — Supplementary Information. [file 41598_2022_6031_MOESM1_ESM.pdf]

Supplementary Information

Aligned CuO nanowire array for a high performance visible light photodetector

Min-Seung Jo[1]◆, Hyeon-Joo Song[2]◆, Beom-Jun Kim[1]◆, Yoo-Kyum Shin[3], Sung-Ho Kim[1], Xu Tian[4], Sang-Min Kim[3], Min-Ho Seo[3, 5], and Jun-Bo Yoon[1]**

[1] School of Electrical Engineering, Korea Advanced Institute of Science and Technology (KAIST), 291 Daehak-ro, Yuseong-gu, Daejeon 34141, Republic of Korea

[2] SAMSUNG ELECTRONICS Co., Ltd. 1, Samsungjeonja-ro, Hwaseong-si, Gyeonggi-do 18448, Republic of Korea

[3] Department of Information Convergence Engineering, College of Information and Biomedical Engineering, Pusan National University, Yangsan, 50612, Republic of Korea

[4] Department of Micro and Nanosystems, KTH Royal Institute of Technology, Brinellvägen 8, 114 28 Stockholm, Sweden

[5] School of Biomedical Convergence Engineering, College of Information and Biomedical Engineering, Pusan National University, Yangsan, 50612, Republic of Korea

E-mail: Min-Ho Seo, mhseo@pusan.ac.kr; Jun-Bo Yoon, E-mail: jbyoon@kaist.ac.kr

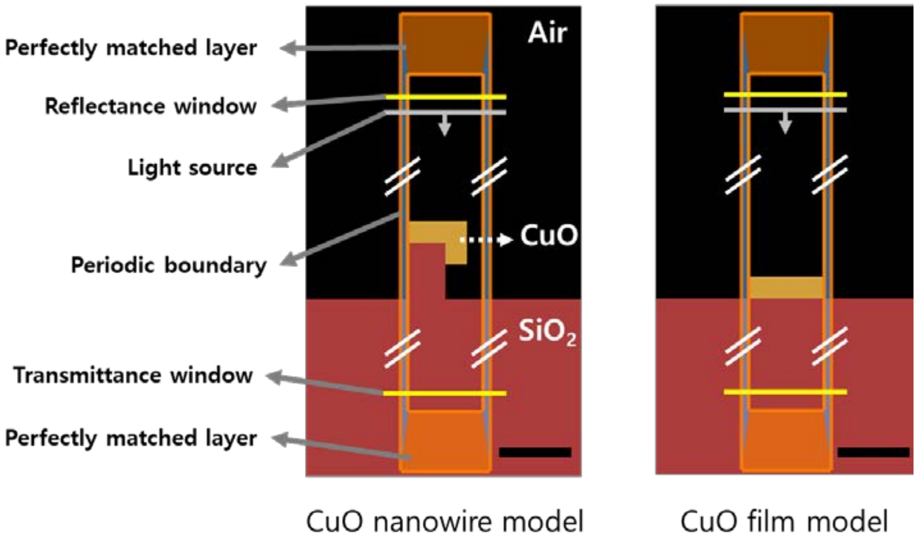

Figure S1. FDTD simulation design for CuO NW (left) and CuO film (scale bar : 400 nm) (Perfectly matched layer : an artificial perfectly absorbing layer for wave equations, Reflectance/Transmittance window : a window getting reflectance and transmittance by calculating the energy going through the window, Light source : the normal incident light with 400~700 nm wavelength, Period boundary : a boundary of FDTD region for calculating a periodic structure.

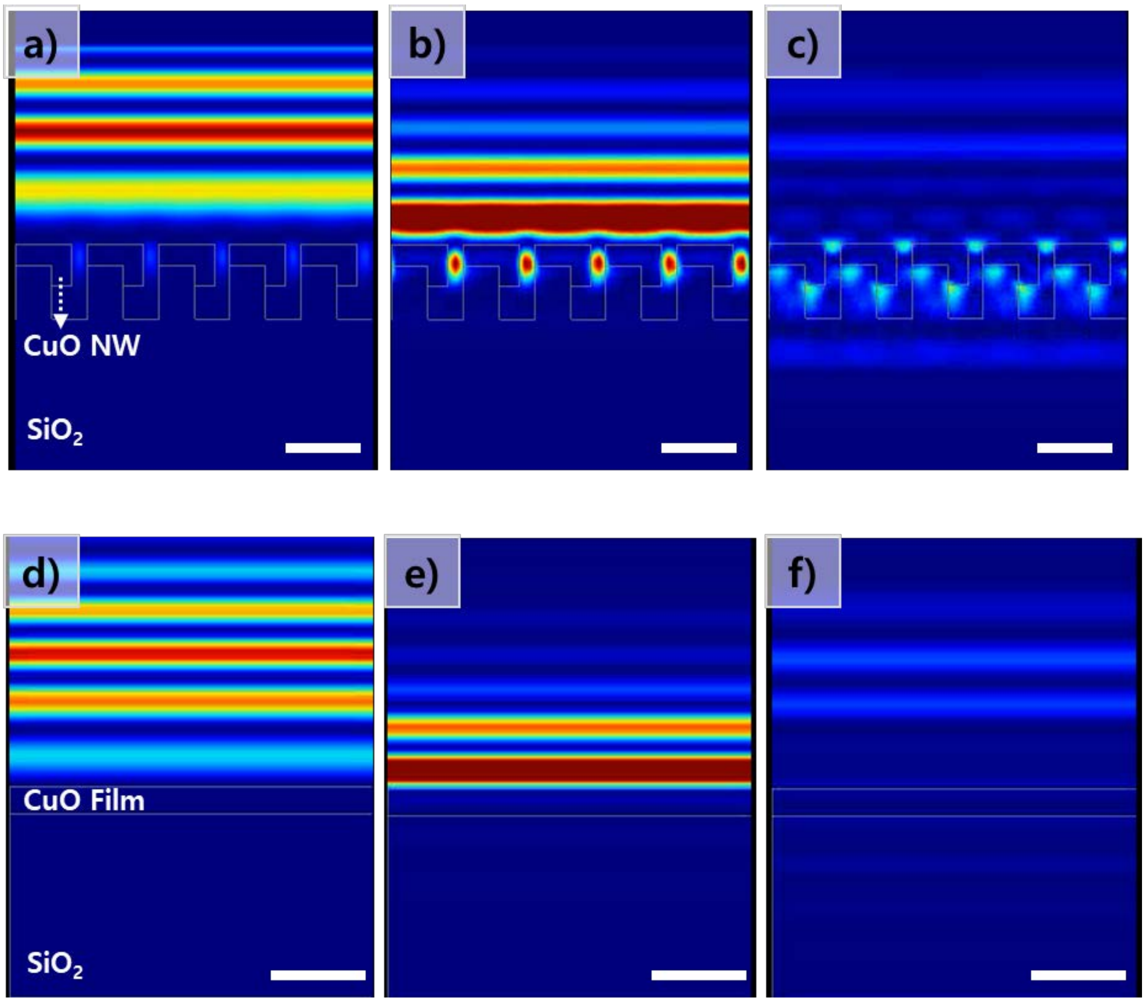

Figure S2. E-field monitoring on the CuO NW and CuO film in time order. a,d) before the incident light reaches to the CuO, b,e) when the incident light reaches to the CuO, c,f) after the incident light reaches to the CuO (scale bar : 500 nm / The wavelength of light source is 400~700 nm).

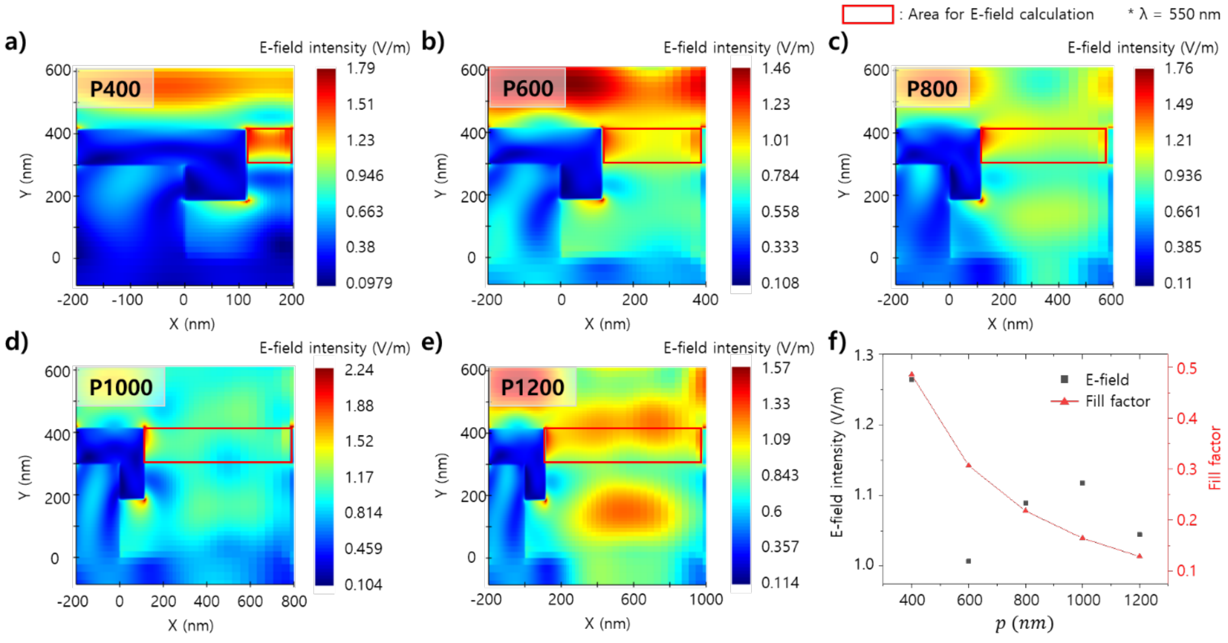

Figure S3. a-e) E-field intensity of the CuO NW with various pitches (p) at 550 nm wavelength of the incident light, f) Average E-filed intensity in the red box between the CuO NWs and the fill factor of CuO in the CuO layer with various pitches.

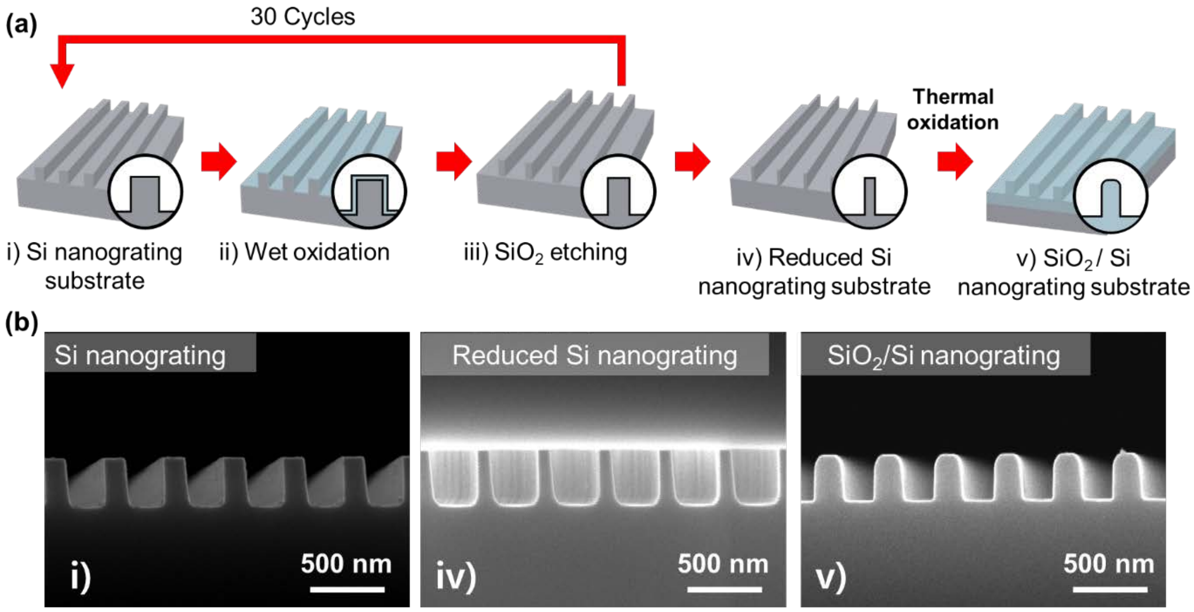

Figure S4. a) The fabrication process of SiO_2 nanograting substrate b) The cross-sectional SEM image of Si nanograting (left; a-i), size reduced Si nanograting (center; a-iv) and oxidized nanograting (right; a-v).

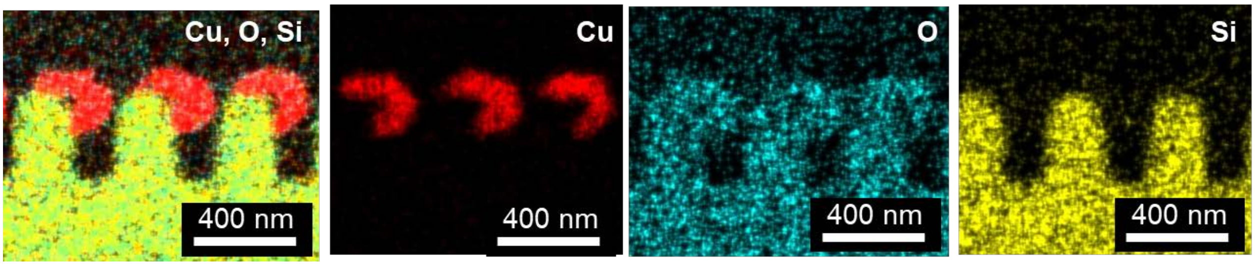

Figure S5. EDS results of the CuO NW on the SiO_2 nanograting.

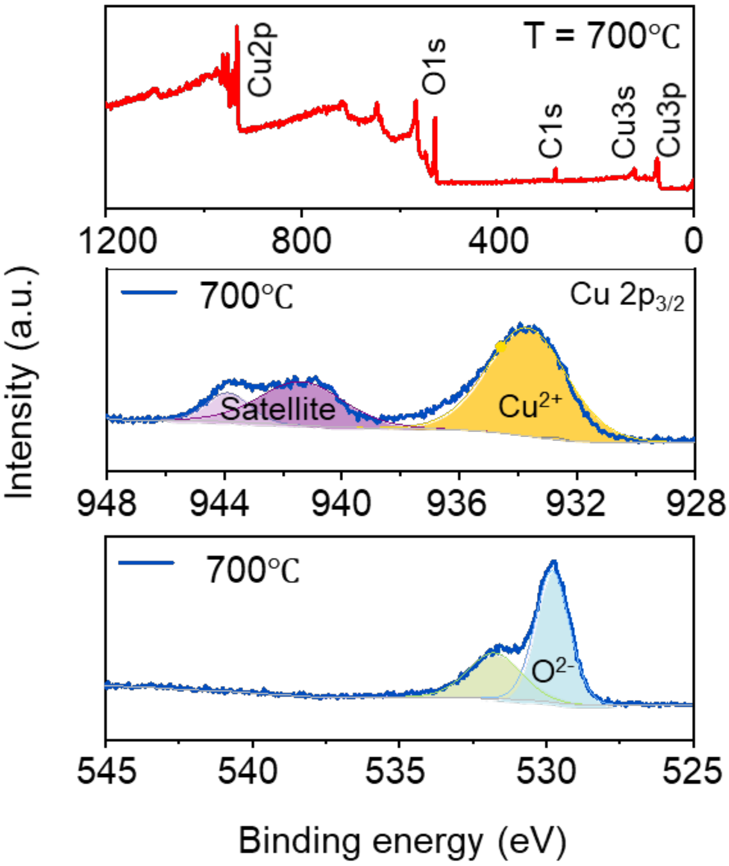

Figure S6. XPS results of the CuO annealed at 700°C.

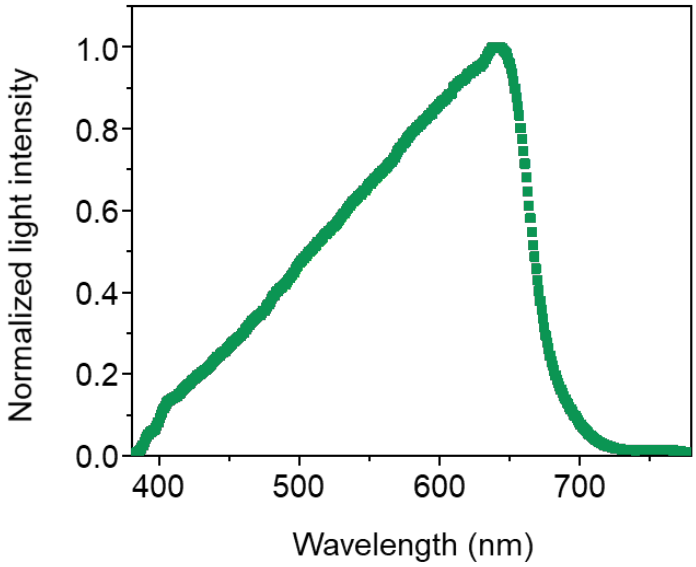

Figure S7. Normalized light intensity of the halogen lamp between 380 nm and 780 nm

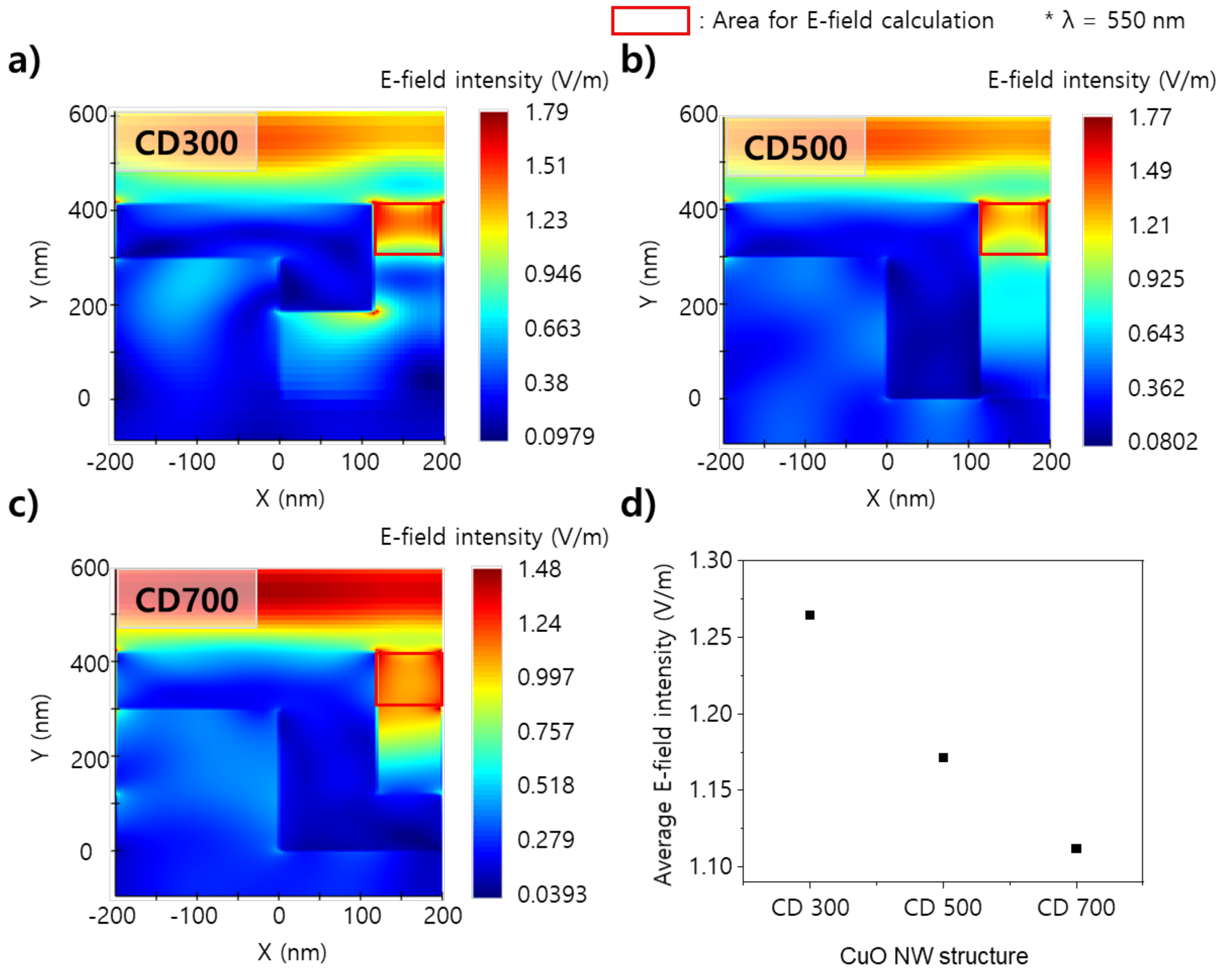

Figure S8. a-c) E-field intensity of the CuO NW with various critical dimensions(*CD*) at 550 nm wavelength of the incident light, d) Average E-filed intensity in the red box between the CuO NW with various *CD*s

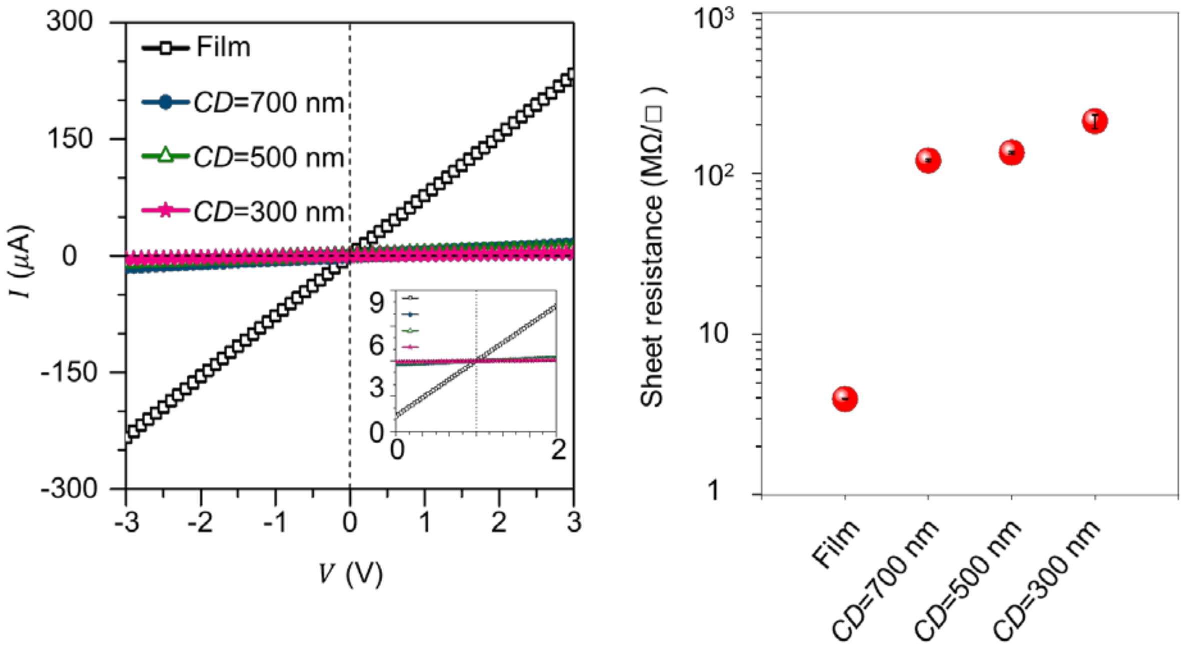

Figure S9. The measured *I-V* curves and sheet resistance of the CuO devices

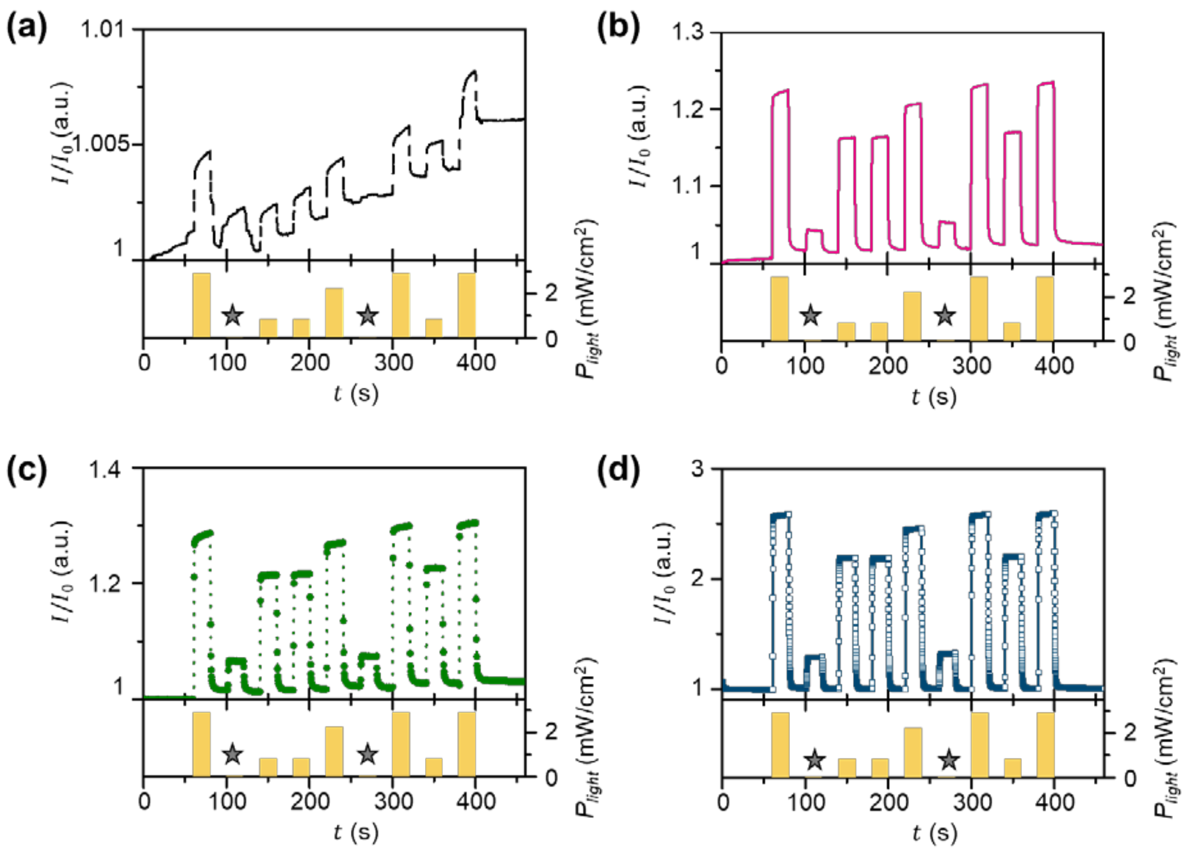

Figure S10. The optoelectronic responses of the CuO devices. a) the CuO film, b) the CuO NW with *CD*=700 nm, c) *CD*=500 nm, d) *CD*=300 nm

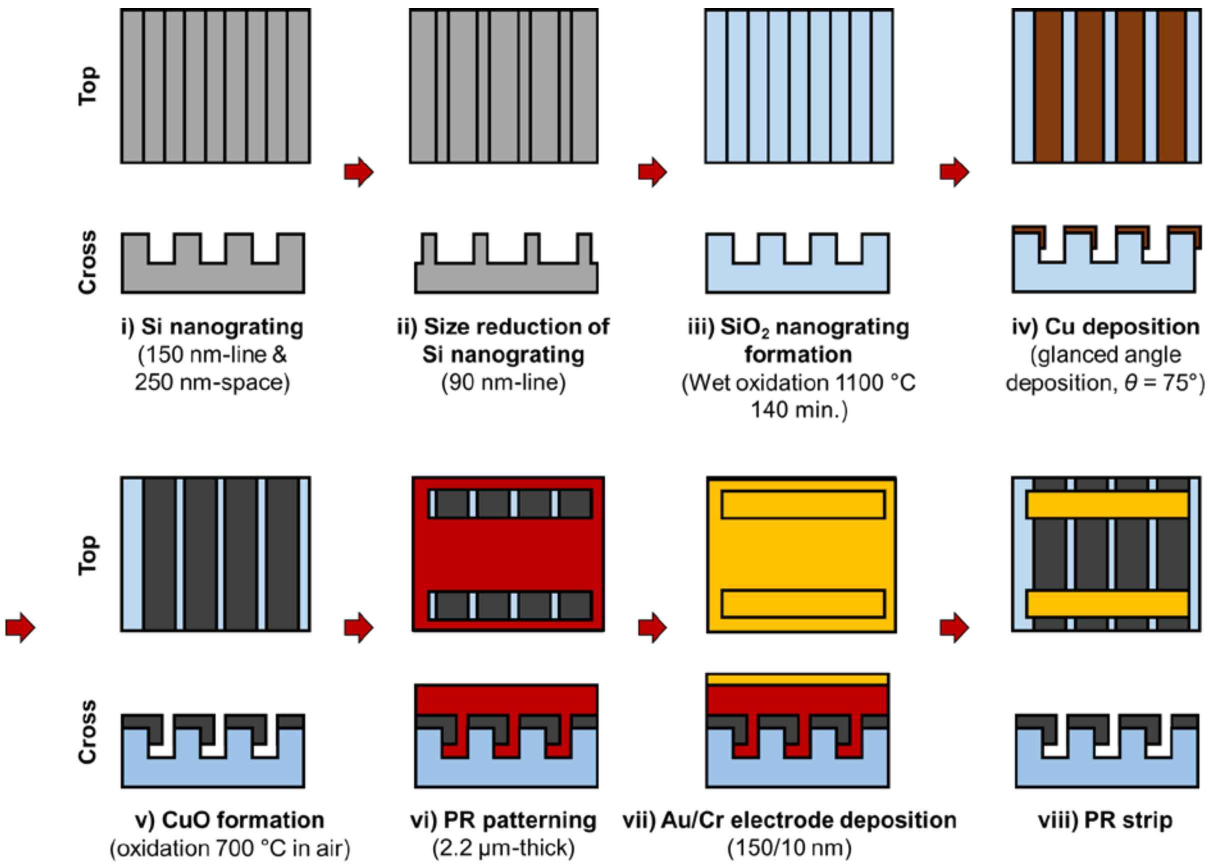

Figure S11. Detailed experimental conditions of CuO photodetectors fabrication process
